# Epidemiology-based analysis of the risks and elimination strategies of the monkeypox outbreak in 2022

**DOI:** 10.3389/fvets.2022.1064766

**Published:** 2022-11-30

**Authors:** Ji-Ming Chen, Rui-Xu Chen, Huan-Yu Gong, Meng-Meng Zhao, Yu-Fei Ji, Ming-Hui Sun, Guo-Hui Li, Su-Mei Tan, Gui-Hong Zhang, Ji-Wang Chen

**Affiliations:** ^1^School of Life Science and Engineering, Foshan University, Foshan, China; ^2^Guangdong Provincial Key Laboratory of Zoonosis Prevention and Control, College of Veterinary Medicine, South China Agricultural University, Guangzhou, China; ^3^Department of Medicine, University of Illinois at Chicago, Chicago, IL, United States

**Keywords:** monkeypox, risk, elimination, epidemiology, outbreak, prediction

## Abstract

Human monkeypox, caused by monkeypox virus, has spread unprecedentedly to more than 100 countries since May 2022. Here we summarized the epidemiology of monkeypox through a literature review and elucidated the risks and elimination strategies of this outbreak mainly based on the summarized epidemiology. We demonstrated that monkeypox virus became more contagious and less virulent in 2022, which could result from the fact that the virus entered a special transmission network favoring close contacts (i.e., sexual behaviors of men who have sex with men outside Africa) and the possibility that the virus accumulated a few adaptive mutations. We gave the reasons to investigate whether cattle, goats, sheep, and pigs are susceptible to monkeypox virus and whether infection with monkeypox virus could be latent in some primates. We listed six potential scenarios for the future of the outbreak (e.g., the outbreak could lead to endemicity outside Africa with increased transmissibility or virulence). We also listed multiple factors aiding or impeding the elimination of the outbreak. We showed that the control measures strengthened worldwide after the World Health Organization declared the outbreak a public health emergency of international concern (PHEIC) could eliminate the outbreak in 2022. We clarified eight strategies, i.e., publicity and education, case isolation, vaccine stockpiling, risk-based vaccination or ring vaccination, importation quarantine, international collaboration, and laboratory management, for the elimination of the outbreak.

## Introduction

**Figure 1:**
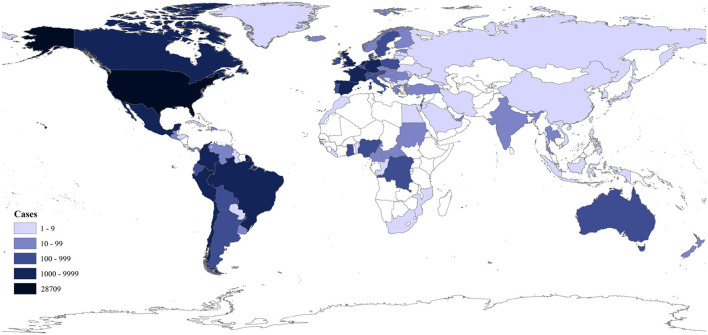
Distribution of the cumulative confirmed human MPX cases in 2022 as of November 7, 2022 as per the data from the CDC (1).

Human monkeypox (MPX) is akin to smallpox and is caused by monkeypox virus (MPXV). MPXV, which mainly circulated in some African countries before 2022, has spread unprecedentedly to more than 100 countries or territories. As of November 8, 2022, 78,379 human cases with 43 deaths have been confirmed (Figure 1) (1–6). The World Health Organization (WHO) declared on July 23, 2022 this multi-country MPX outbreak a public health emergency of international concern (PHEIC) (3). The outbreak was abbreviated as the 2022 outbreak below.

MPXVs are typical poxviruses with large and brick-shaped enveloped virions with a lipoprotein outer membrane. Its double-stranded DNA genome is approximately 190 kb and encodes about 200 proteins. MPXVs constitute the species *MPX virus* and share the same genus *Orthopoxvirus*, subfamily *Chordopoxvirinae*, and family *Poxviridae* with *Abatino macacapox virus, Akhmeta virus, Camelpox virus, Cowpox virus* (CPXV), *Ectromelia virus, Raccoonpox virus, Skunkpox virus, Vaccinia virus* (VACV), *Variola virus* (VARV, the etiology of smallpox), *Taterapox virus*, and *Volepox virus* (7).

Here we summarized the epidemiology of MPX through a literature review and elucidated the risks and elimination strategies of the 2022 outbreak mainly based on the summarized epidemiology.

## Epidemiology of human MPX

In temporal and spatial distribution, MPX is endemic in central and western Africa. MPXV and its infection in animals (cynomolgus monkeys) were first identified in 1958 (2). MPXV infections in humans, which were first identified in 1970, increased significantly in endemic African regions in recent decades (8), likely due to the decline of population immunity against smallpox with the cessation of smallpox vaccination in the 1980's (9). Human cases are often found close to tropical rainforests, where there are animals that carry the virus (3, 10). In recent years, thousands of humans were infected with MPXV annually in the endemic African regions (5), and a few imported human cases outside Africa were identified after 2017 (8). In 2003, imported pet rodents spread the virus to pet prairie dogs in the USA and sparked a small-scale autochthonous human MPX outbreak (11). As of November 7, 2022, 86.6% (67,859/78,379) of confirmed human cases in the 2022 outbreak were reported from the following ten countries in Americas or Europe: the USA (28,709), Brazil (9,312), Spain (7,336), France (4,097), the United Kingdom (3,701), Germany (3,668), Colombia (3,523), Peru (3,204), Mexico (2,901), and the Netherlands (1,237) (Figure 1) (1).

In host distribution, MPXV can infect various mammals, including rodents (e.g., squirrels, rats, mice, giant-pouched rats, dormice, Gambian giant rats, chinchillas, marmots, groundhogs, and prairie dogs), primates (e.g., monkeys, apes, humans), carnivores (e.g., dogs), lagomorphs, and insectivores (e.g., hedgehogs, shrews) (1, 3, 12–14). Because MPXV can infect various species of mammals (1, 3), it could infect cattle, goats, sheep, and pigs, which should be tested in experiments. An experiment showed that MPXV replicated in pig skin (15).

In clinical epidemiology, the incubation period in humans is 5–21 days, and symptoms last 2–4 weeks (1, 3, 8). Early symptoms of infected humans include fever, headache, fatigue, and muscle pains, like influenza (3). Typical skin lesions (rashes) usually appear on the face, then on the trunk, and then on other sites (e.g., hand palms) (1, 3). Many male cases in the 2022 MPX outbreak presented with genital and peri-anal lesions (1). Patients can encounter secondary infections, pneumonia, encephalitis, sepsis, and vision impairment (3, 16). Some infected animals and humans are asymptomatic (6, 17). For example, three human MPX cases in Belgium identified in 2022 were asymptomatic (17). Animal experiments suggested that infection routes affect clinical severity (18). For example, it was found that nasal inhalation is more fatal than other inoculation routes in white rats, CAST/EiJ mice, and prairie dogs (13, 19, 20). This may be because inflammation is more dangerous in the lungs than in other organs (21). Most human MPX cases before 2022 occurred in children in the endemic regions (8), but most human cases in the 2022 outbreak were young males unvaccinated against smallpox (1). For example, of the first 23,667 confirmed human MPX cases in the USA in the 2022 outbreak, 0.18% were <16 years of age, 88.16% 16–65 years of age, 0.56% >65 years of age, 94.64% were men, 2.20% women, 2.17% transgender women, 0.30% transgender men, 0.69% another sex/gender, 90.34% ≤ 50 years of age (usually unvaccinated against smallpox as smallpox vaccination terminated in the USA in 1972), and 9.67% >50 years of age (usually vaccinated against smallpox vaccines) (1).

**Figure 2:**
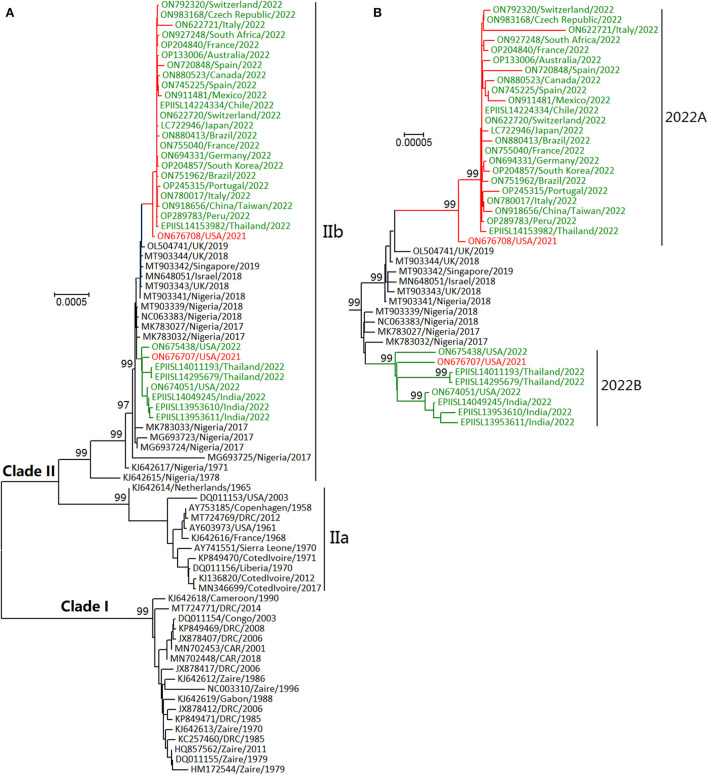
Phylogenetic relationships of some monkeypox viruses (MPXVs) during the years 1958–2022 **(A)** and in recent years **(B)**. Those MPXVs in 2022 shown in green letters were classified into two lineages (2022A and 2022B), which were similar to the two MPXVs detected in the USA in 2021 shown in red letters.

In phylogenetic distribution, as shown in Figure 2, MPXVs were classified into Clades I and II (3, 6, 8). Clade I (formerly designated as “Central African” or “Congo Basin” clade) circulates in central Africa (Cameroon, the Democratic Republic of the Congo (DRC), and Central African Republic (CAR), Gabon, and the Republic of the Congo). Clade II (formerly designated “West African” clade) mainly circulates in western Africa (Benin, Cameroon, Cote d'Ivoire, Liberia, Nigeria, and Sierra Leone). MPXV could have originated 3500 (95% HPD 2200–5400) years ago from some cowpox viruses and evolved into two clades about 600 (95% HPD 300–1,000) years ago (22, 23). Clade II includes Clades IIa and IIb, which likely diverged in the 1970's (24). Furthermore, two lineages of MPXVs within Clade IIb circulated in multiple countries in 2022 (Figure 2) (24). Of known orthopoxviruses except some CPXVs, VACVs are the closest in phylogenetics to MPXVs, and vice versa (24). The phylogenetic relationships in Figure 2 were calculated using the software MEGA (version X) (25), the neighbor-joining method, the maximum composite likelihood model, and some viral genomic sequences (the sequences were designated with their GenBank accession numbers, the sample-collection regions, and the sample-collection years; rates among sites were treated in gamma distribution; gaps were treated as partial deletion; bootstrap values were calculated with 1,000 replicates).

**Figure 3:**
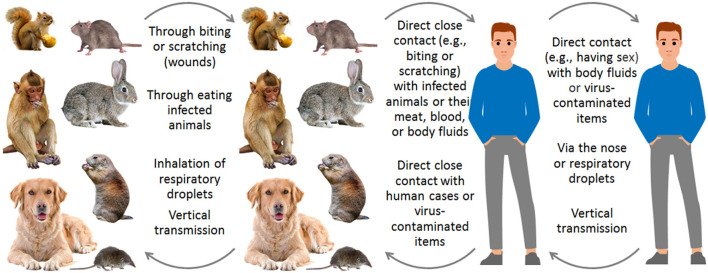
Transmission routes of MPX in animals and humans.

In transmission routes, MPXVs spread among animals and humans through three routes (1, 3, 6, 8, 26): direct close contact (e.g., sexual activity and biting) with infected humans or animals or their wounds, meat, blood, body fluids, or virus-contaminated items; inhalation of respiratory droplets; vertical transmission during pregnancy (Figure 3). In the 2022 outbreak, MPXV mainly circulates in men who have sex with men (MSM) through sexual behaviors (1, 3, 4), and a human with prolonged seminal viral shedding was identified (27).

In infection courses, usually, human MPXV infection is assumed to be transient, but we suspect that the infection in primates could sometimes be latent, as suggested by two studies. One was in 1958 in Denmark, where MPX occurred in two batches of cynomolgus monkeys only when they had been transported to Copenhagen for 51 and 62 days (2). The other was in 1961 in the USA, where MPX occurred in a cynomolgus monkey only after the monkey had been maintained in the same room for 11.5 months and the monkey was irradiated 45 days before the onset of MPX (28).

**Figure 4:**
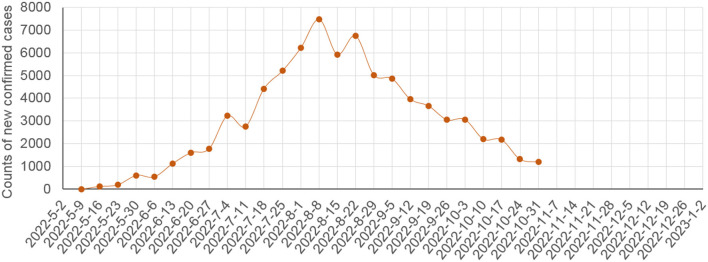
Counts of global weekly new confirmed human MPX cases in the 2022 outbreak as per the data from the WHO (29).

In contagiousness, MPX has become more contagious in humans in recent decades in their endemic regions, because in the 1970's and 1980's, more than 70% of human cases were infected *via* the animal-to-human route, whereas in recent years, more than 70% of human cases were infected *via* the human-to-human route (8). Nevertheless, the reproduction number (R_0_) value of MPXV before 2022 was <1.0 (6, 8), because many events of human-to-human transmission terminated naturally in endemic regions. However, mathematically, this value should be >1.0 in the 2022 outbreak before the outbreak was declared a PHEIC by the WHO with the rapid increase in human cases lasting for months (Figure 4). For example, the R_0_ value was 1.82–3.26 among MSM in Italy (30). The significant increase in the R_0_ value could result from the fact that the virus entered a special transmission network favoring close contacts (i.e., sexual behaviors of MSM) outside Africa (31, 32). It also could result from the possibility that MPXV accumulated a few adaptive mutations in 2022 (33).

In the case fatality rate (CFR), we assumed that the outcome of a human MPX case would be known on the 21^st^ day after the case is reported (1, 3, 4). Then, the outcomes of the first 52,994 human cases confirmed before September 5, 2022 were known on September 26, 2022 (26 deaths and 52,968 recoveries). Therefore, the CFR of the 2022 outbreak was approximately 0.049%. This value is much lower than the CFRs of smallpox (approximately 30%), Clade I (approximately 10.6%) before 2022, and Clade II (approximately 3.6%) (8). The significant decline in the CFR of MPX in 2022 could result from better medical services outside Africa than in the endemic African regions or from the possibility that the virulence of MPXV could have declined in 2022.

## Risk of the 2022 outbreak

In risk factors, as per the epidemiology of MPX elucidated above, endemic countries, international travel, importation of mammals, humans unvaccinated with smallpox vaccines, MSM, crowded parties, close contact with infected humans, family members of infected humans, the trash of infected humans, healthcare workers in affected regions, vaccine unavailability, squirrels, rodents, non-human primates, pet animal markets or traders, and zoos are all risky for the transmission of MPX (1–6, 8–20). Forest regions, particularly those with squirrels and oil palms, were positively associated with annual MPX incidence (10, 34).

In future prediction, Figure 4 showed that, before the WHO declared the 2022 outbreak a PHEIC on July 23, 2022, the counts of global weekly new MPX cases increased rapidly, and the counts declined rapidly after August 8, 2022 (i.e., within one maximum incubation period of human MPX after the declaration). Therefore, the control measures strengthened worldwide after the declaration [e.g., the publicity regarding MPX was strengthened globally due to the declaration, and hence many MSM knew MPX and its risks, minimized their risky sexual behaviors, and actively participated in the ring vaccination (1)], could have been highly effective to control the outbreak in 2022. Figure 4 suggests that the 2022 outbreak could be eliminated before 2023 if the current control measures will be maintained or strengthened.

If we looked at the counts of new human cases shown in Figure 4 rapidly rising before the WHO declaration of the PHEIC on July 23, 2022, the future of the 2022 outbreak could exhibit one of the potential scenarios listed in Table 1, depending on the implemented control measures and the genomic changes of MPXV. In scenarios A–D, the 2022 outbreak will be eliminated outside Africa before the case count reaches 300,000, 1,000,000, 10,000,000, and >10,000,000, respectively. In scenarios E and F, the 2022 outbreak becomes endemic outside Africa without or with increased transmissibility or virulence. Scenarios D–F could represent a disastrous pandemic. Scenario F with natural wildlife reservoirs could be more intractable than smallpox, which circulated in humans without natural reservoirs. Under scenario F, almost all people who have not been vaccinated against smallpox should be vaccinated against MPX, which could cost worldwide more than 530 billion USD in 10 years: 400 billion USD [more than 4 billion people younger than 42 years of age (who have not been vaccinated against smallpox) multiplied by 50 USD/dose and 2 doses/person] plus 13 billion USD (0.13 billion newborns/year multiplied by 50 USD/dose and 2 doses/newborn) each year in the 10 years. Fortunately, Figure 4 suggests that scenario A in Table 1 is promising if the current control measures will be maintained or strengthened.

**Table 1:** Future potential scenarios, relevant factors, and elimination strategies of the monkeypox outbreak in 2022.

**Aspect**	**Detail**
Future potential scenarios	A: elimination outside Africa with <300,000 cases totally
	B: elimination outside Africa with <1,000,000 cases totally
	C: elimination outside Africa with <10,000,000 cases totally
	D: elimination outside Africa with >10,000,000 cases
	E: endemicity outside Africa without increased transmissibility or virulence
	F: endemicity outside Africa with increased transmissibility or virulence
Factors useful to eliminate the outbreak	A: the control measures have been strengthened worldwide after the WHO declared the outbreak a PHEIC on 23 July 2022
	B: most monkeypox infections in humans are likely self-restricted
	C: MPXV infection is relatively easy to be suspected
	D: monkeypox vaccination and infections usually lead to robust immunity
	E: monkeypox virus mutates relatively slowly
	F: monkeypox virus spreads relatively slowly
	G: monkeypox vaccines have been ready for mass production
	H: the global weekly new monkeypox cases have been rapidly declining
Factors impeding the elimination of the outbreak	A: monkeypox virus has infected too many people in many countries
	B: monkeypox virus can infect various species of domestic and wild mammals
	C: the viral transmissibility or virulence can be increased due to gene mutations
	D: monkeypox could not have aroused enough vigilance due to its low virulence
	E: bioterrorism attacks can accelerate the virus spread
	F: some countries cannot eliminate monkeypox promptly

Multiple factors aid the world in eliminating the 2022 outbreak (Table 1). The WHO declaration of the PHEIC facilitated global publicity and education about MPX and its risks. Unlike human immunodeficiency virus (HIV) infections, most MPXV infections in humans are likely self-restricted (1, 3, 4, 16). Unlike various infectious diseases (e.g., viral hepatitis, viral encephalitis, and coronavirus infection), MPXV infection is relatively easy to be suspected because of its skin lesions and clinical epidemiology (i.e., most human MPX cases occur in young MSM in the 2022 outbreak), although confirmation of MPX cases relies on laboratory detection. Unlike RNA viruses, MPXV is a DNA virus and mutates relatively slowly, and hence, MPX vaccines (e.g., the JYNNEOS or ACAM2000 vaccines produced in the USA) do not need frequent updates. MPX spreads more slowly than COVID-19 and currently mainly circulates in MSM, although some children and women have been infected through close direct or indirect contact (1, 3, 4). Moreover, MPX vaccines have been ready for mass production in some countries, because smallpox vaccines (e.g., those made of live attenuated VACVs) can be directly used for the prevention of MPX, as suggested by various epidemiological studies and animal experiments (1, 6, 9, 35). Smallpox vaccination imparted approximately 85% protection against monkeypox (6, 9). In the 2022 outbreak, unvaccinated people in the USA had 14 times the risk of MPX compared to people who were vaccinated (1).

This is also supported by the fact that MPXV and VACV are close to each other in phylogenetics (6, 24). Unlike vaccination and infection with influenza or COVID-19, vaccination and infection with MPXV probably lead to long-term robust immunity (1, 6, 8, 9, 35). Collectively, it is easier for the world to eliminate the 2022 outbreak outside Africa than to eliminate a large COVID-19 outbreak, and the global weekly new MPX cases have been rapidly declining (Figure 4).

On the other hand, multiple factors hinder the world from eliminating the 2022 outbreak (Table 1) (32).

First, MPXV has infected too many people in many countries in 2022 (Figure 1), and these infected people could transmit MPXV to other people and provide opportunities for the virus to accumulate adaptive mutations in humans.

Second, MPXV can infect various species of domestic and wild animals (1–3, 6). Therefore, infected humans can spread MPXV to domestic or wild animals outside, which is supported by the fact that a domestic dog was infected with MPXV by an infected human in 2022 (36). Wild animals can be infected by contacting MPXV-contaminated rubbish of infected humans or by direct close contact with infected domestic animals or their meat, blood, body fluids, or possibly excrement (1). The 2022 outbreak could thus lead to endemicity outside Africa.

Third, the contagiousness of MPXV in humans could increase through genomic mutation. If MPXV accumulates some adaptive mutations and replicates more efficiently in humans, it could be more contagious and virulent in humans simultaneously, as per the trade-off theory regarding the long-term evolution of virulence of pathogens (37). This possibility is supported by the fact that smallpox, goatpox, and lumpy skin disease are all highly virulent and highly contagious in their hosts (38). Previous studies showed that clade I MPXV is more virulent and more contagious in humans than clade II MPXV (6).

Fourth, the 2022 outbreak could not have aroused the vigilance of many threatened human populations worldwide because of its low CFRs (approximately 0.049% as mentioned above). This could lead to multiple waves of human MPX infections in the coming years.

Fifth, the 2022 outbreak can provide live MPXV strains for laboratory engineering and bioterrorism attacks, which can accelerate the virus′ spread worldwide (39). This is indicated by an international official exercise conducted in 2021, which showed that an engineered MPXV released by bioterrorists could cause 3.2 billion human infections and 270 million deaths (40).

Sixth, some countries, particularly those in war or severe poverty, cannot eliminate human MPX infections promptly. They cannot identify and isolate human MPX cases promptly, and they have not stocked enough smallpox or MPX vaccines and antivirals. The global vaccine inequity and antiviral inequity that facilitated the spread of SARS-CoV-2 could be repeated with MPX (31).

## Strategies to eliminate the 2022 outbreak

As per the epidemiology and risks given above, the following strategies should be considered to eliminate the 2022 outbreak outside Africa earlier (Table 2).

**Table 2:** Strategies to eliminate the monkeypox outbreak in 2022.

**Strategy**	**Rationale**
Publicity and education	To make people know the disease risks, how to avoid the risks, how to report suspected cases, and how to control and eliminate the infection
Case isolation	To block the disease transmission from infection sources
Vaccine stockpiling	To prepare enough vaccines to protect those at high risk and block the disease transmission
Epidemiological investigation	To identify or exclude new human cases, new animal cases, and new risks promptly, to block the disease transmission
Risk-based vaccination	To minimize human infections and block the disease transmission from susceptible persons
Importation quarantine	To minimize the transboundary transmission of the disease through infected animals or humans
International collaboration	To aid low- and middle-income countries in eliminating the 2022 outbreak by sharing epidemiological information and vaccines
Laboratory management	To avoid laboratory spill-over events and reduce the relevant bioterrorism risk

First, all countries should strengthen publicity and education regarding the risks, prevention, treatment, and control of the disease, preferably through new media, which are more efficient in communication and more capable of avoiding stigmatization than traditional media. All people should know how to report suspected human or animal cases of MPX to the government, public health agencies, hospitals, etc. Likely due to strengthened publicity and education, MSM in the USA have taken steps to protect themselves and their partners from MPX (48% of them reduced the number of sex partners, 50% reduced one-time sexual encounters, and 50% reduced sex with partners met on dating apps or at sex venues) (1).

Second, infected people should be isolated until they are no longer infectious, and their rubbish should be properly treated, to cut off the transmission of MPXV in humans and animals.

Third, MPX vaccines should be well stockpiled worldwide. The USA decided to stockpile 13 million doses of MPX vaccines in May 2022. The vaccines stockpiled worldwide can be employed in endemic African countries if the 2022 outbreak is eliminated in the near future.

Fourth, epidemiological investigation of humans and animals living in threatened areas should be strengthened in order to identify new human or animal cases promptly. As explained above, it is desirable to investigate whether cattle, goats, sheep, and pigs are susceptible to MPXV, which is important for the elimination of the 2022 outbreak as they are populous and frequently in close contact with humans. It is also desirable to investigate whether MPXV infection could be latent or persistent in some primates and humans infected with HIV.

Fifth, if possible, family members, sexual partners, healthcare workers, and other close contacts of human MPX cases should be vaccinated imminently (ring vaccination) to block the transmission in affected regions (41). If possible, all people unvaccinated against smallpox can be vaccinated voluntarily, which could enhance the vaccination coverage of MSM without stigmatization.

Sixth, the quarantine of imported animals should be strengthened. This is supported by the fact that pet rodents imported from Ghana spread the virus to pet prairie dogs in the USA in 2003, which further infected more than 70 people (5).

Seventh, robust international collaboration in sharing epidemiological information and vaccines is needed to aid low- and middle-income countries to eliminate the 2022 outbreak (42). Mass vaccination in endemic African countries can not only protect people in these countries but also reduce the possibility of MPX spreading to other countries again in the future (43).

Eighth, all countries should consider strictly restricting laboratory research with live MPXV to avoid laboratory spill-over events (39, 40).

## Conclusions

Here we summarized the epidemiology of MPX through a literature review and elucidated the risks and the elimination strategies of this outbreak mainly based on the summarized epidemiology. We demonstrated that MPXV became more contagious and less virulent in 2022, which could result from the fact that the virus entered a special transmission network favoring close contacts outside Africa and the possibility that the virus accumulated a few adaptive mutations. We gave the reasons investigating whether cattle, goats, sheep, and pigs are susceptible to MPXV and whether infection with MPXV could be latent in some primates. We listed six potential scenarios for the future of the outbreak and multiple factors aiding or impeding its elimination. We showed that the control measures strengthened worldwide after the WHO declared the outbreak a PHEIC could eliminate the outbreak in 2022. We listed eight strategies for the elimination of the 2022 outbreak. Collectively, this analysis provides novel insights into the risks and elimination of the 2022 outbreak.

## Author contributions

J-MC conceived, designed and supported this study, analyzed the data, and drafted the manuscript. G-HZ and J-WC made the core conclusion, analyzed the data, and revised the manuscript. R-XC, H-YG, M-MZ, M-HS, Y-FJ, G-HL, and S-MT collected and analyzed relevant data and revised the manuscript. All authors contributed to the article and approved the submitted version.

## Funding

This study was supported by the Open Fund of Guangdong Provincial Key Laboratory of Zoonosis Prevention and Control (No. ZMM2021) and the Open Competition Program of Top-Ten Critical Priorities of Agricultural Science and Technology Innovation for the 14th Five-Year Plan of Guangdong Province (2022SDZG02).

## Conflict of interest

The authors declare that the research was conducted in the absence of any commercial or financial relationships that could be construed as a potential conflict of interest.

## Publisher's note

All claims expressed in this article are solely those of the authors and do not necessarily represent those of their affiliated organizations, or those of the publisher, the editors and the reviewers. Any product that may be evaluated in this article, or claim that may be made by its manufacturer, is not guaranteed or endorsed by the publisher.
